# A Potent Lignan from Prunes Alleviates Inflammation and Oxidative Stress in Lithium/Pilocarpine-Induced Epileptic Seizures in Rats

**DOI:** 10.3390/antiox9070575

**Published:** 2020-07-02

**Authors:** Fadia S. Youssef, Esther T. Menze, Mohamed L. Ashour

**Affiliations:** 1 Department of Pharmacognosy, Faculty of Pharmacy, Ain Shams University, Cairo 11566, Egypt; fadiayoussef@pharma.asu.edu.eg; 2 Department of Pharmacology and Toxicology, Faculty of Pharmacy, Ain Shams University, Abbassia, Cairo 11566, Egypt; esther.menze@gmail.com

**Keywords:** anti-inflammatory, epilepsy, oxidative stress, pinoresinol-4-*O*-*β*-d-glucopyranoside, pilocarpine, *Prunus domestica*

## Abstract

*Prunus domestica* L. is an edible plant that is included in the family Rosaceae and proven to possess potent anti-inflammatory and anxiolytic activity. Pinoresinol-4-*O*-β-d-glucopyranoside (PGu) was isolated from *Prunus domestica* methanol extract and its structure was determined using 1-D and 2-D NMR (one- and two-dimensional nuclear magnetic resonance). PGu was evaluated for its anticonvulsant activity using lithium/pilocarpine-induced epileptic seizures in rats. PGu displayed a notable antioxidant and anti-inflammatory activity in vitro. It ameliorates the seizures triggered by pilocarpine in a dose-dependent manner, manifested by retarding seizure onset, reducing the number of rats developing seizures, and enhancing the survival of animals after seizure exposure. PGu reduced MDA (malondialdehyde) level by 24.2% in addition to increasing catalase activity by 44.4% at 50 mg/kg b.w compared to pilocarpine-treated animals. This was confirmed by histopathological examination in which pretreatment with PGu (50 mg/kg b.w.) attenuated neurodegeneration and seizures with no histopathological alteration in neurons of the cerebral cortex. In the immunohistochemical examination, it significantly declined the elevated Cyclooxygenase-2 (COX-2) by 40% and decreased Inducible nitric oxide synthase (iNOS) expression by 18% as expressed by the optical density. PGu revealed a pronounced fitting within the active site of 5-LOX (lipoxygenase-5) with a free binding energy (∆G) equals to −65.05 kcal/mol. PGu could perfectly serve as a potent lead drug for the relief of epileptic seizures, which appeals to many patients owing to its natural origin.

## 1. Introduction

Epilepsy is one of the popular neurological dysfunctions, which is characterized by the recurrent occurrence of unprovoked seizures caused by hyperneuronal activity and a sudden rush of electrical activity in the brain [1]. Unfortunately, it affects nearly 60 million people all over the globe [2]. It could be triggered by injuries, such as head injury and hypoxia, or by status epilepticus. The latter is induced by the overproduction of reactive oxygen and nitrogen species that immediately precedes oxidative stress, inflammation, and tissue damage. Basically, the vigorous liberation of cytokines as well as interleukins and their existence in the tissue is accompanied by functional impairment in the neurons. Furthermore, reactive oxygen species (ROS) and nitric oxide synthase (NOS) can contribute to neuronal damage and neuroinflammation, which are observed in status epilepticus. Increased neuronal hyperexcitability triggered by reduced Gamma aminobutyric acid (GABA) function and enhanced *N*-methyl-d-aspartate (NMDA) receptor activity causes massive Ca++ entry in cells, which results in seizures, with subsequent generation of ROS [3,4].

Many antiepileptic synthetic agents, such as carbamazepine, oxcarbazepine, ethosuximide, felbamate, phenobarbital, as well as valproic acid and phenytoin, are present. Although they can effectively ameliorate epileptic seizures in the majority of patients, a considerable percentage, estimated by 30% to 40%, still suffer from pharmacoresistance during the course of their therapy. In addition, these synthetic agents trigger undesirable side effects [5]. The plant kingdom provides an everlasting source of naturally occurring drugs and phytoconstituents that effectively participate in the amelioration of epileptic seizures. Herbal drugs have been highlighted, particularly in traditional medicine, and display significant antiepileptic results with relatively minimal side effects [6,7].

*Prunus domestica* L. is an edible plant that is included in the family Rosaceae. It is native to western Asia, mainly in the Caucasus area. Its edible fruits have shown a high efficacy in relieving leucorrhoea as well as controlling abnormal menstruation and combating oxidative stress owing to their high content of polyphenols. Recently, *Prunus domestica* has been proven to possess potent anti-inflammatory and anxiolytic activity [8].

In this context, pinoresinol-4-*O*-β-d-glucopyranoside, a lignan glycoside, was isolated from *Prunus domestica* total methanol extract. This study aimed to correlate *Prunus domestica* anti-inflammatory and anxiolytic activity to a compound rather than chlorogenic acid, which was previously reported to possess an anti-inflammatory effect. Pinoresinol-4-*O*-β-d-glucopyranoside, was evaluated for its antioxidant and anti-inflammatory activity in vitro using 2,2-diphenyl-1-picrylhydrazyl (DPPH) scavenging capacity and lipoxygenase inhibitory activity assays, respectively. Moreover, the ability of pinoresinol-4-*O*-β-d-glucopyranoside to counteract epileptic seizures was estimated in vivo using lithium/pilocarpine-induced status epilepticus in rats, which was further ascertained via histopathological and immunohistochemical evaluation. In addition, in silico molecular modelling was done in the 5-lipoxygenase (5-LOX) active site to confirm the anti-inflammatory activity by visualizing the possible interactions at the binding site.

## 2. Materials and Methods

### 2.1. Chemicals and Drugs

Pilocarpine hydrochloride was obtained from Alexandria Co. for pharmaceuticals and chemical industries, Egypt. LiCl was purchased from El-Gomhouria for trading chemicals, Cairo, Egypt. Atropine sulfate was obtained from Sigma-Aldrich Co., St. Louis, MO, USA. Spectrophotometric diagnostic kits for the assessment of malondialdhyde (MDA) level as well as catalase activity were purchased from (Biodiagnostics, Cairo, Egypt). All other consumed chemicals were of the highest grade.

### 2.2. Plant Material

*Prunus domestica* L. (Rosaceae) dried fruits were obtained from the Egyptian market. Identification and authentication were performed morphologically by one of the authors (M.L.A.), Associate Professor of Pharmacognosy, Faculty of Pharmacy, Ain Shams University. A voucher specimen was kept at Pharmacognosy Department, Faculty of Pharmacy, Ain Shams University, Egypt with the code PHG-P-PD-254.

### 2.3. Preparation of the Plant Extract

Dried prunes (7 kg) were extracted at 25 °C with (3 × 7 L) distilled methanol till exhaustion followed by filtration and evaporation under vacuum by a rotary vacuum evaporator at 40 °C. A semisolid residue (1.7 kg) was obtained and then fractionated using a Diaion HP-20 packed column chromatography employing three solvent systems, which were water, methanol, and acetone.

### 2.4. Isolation and Identification of Pinoresinol-4-O-β-d-glucopyranoside

#### 2.4.1. Isolation of Pinoresinol-4-*O*-β-d-glucopyranoside

About 30 g of the methanol fraction (PMF) were further fractionated using a polyamide 6S packed column using water/methanol of decreasing polarity till 100% pure methanol was reached to give 10 main fractions. Fraction II was consequently fractionated and purified on a normal phase silica gel packed column using dichloromethane/methanol of increasing polarity, where 160 mg of pinoresinol-4-*O*-β-d-glucopyranoside (PGu) were obtained with 90:10 (CH_2_Cl_2_/MeOH) as a solvent system.

#### 2.4.2. Identification of Pinoresinol-4-O-β-d-glucopyranoside

##### Nuclear Magnetic Resonance Spectroscopy (NMR)

A Varian 500 MHz AC NMR spectrometer was used to perform ^1^H and ^13^C (APT) NMR analyses using 500.13 and 125.67 MHz as the operating frequencies, respectively. The isolated compound was solvated in deuterated dimethyl sulfoxide (DMSO) ((Deutero, Kastellaun, Germany) and δ ppm was used to express the chemical shift relative to the solvent.

##### Pinoresinol-4-*O*-β-d-glucopyranoside

**Figure 1:**
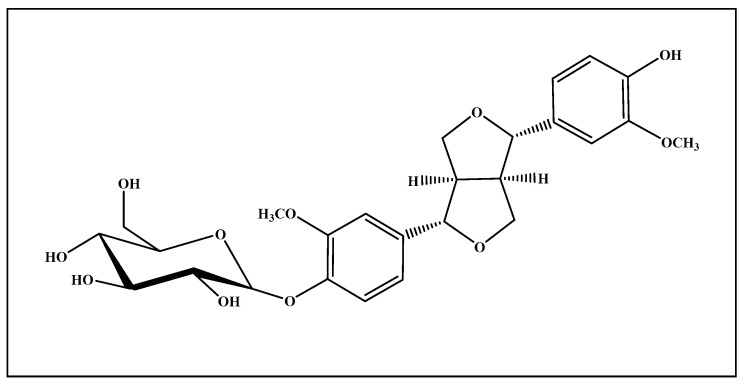
Pinoresinol-4-*O*-*β*-d-glucopyranoside (PGu) structure.

It was isolated as a very pale yellow almost white powder (Figure 1). Its ^1^H- NNR (500 MHz) and ^13^C-NNR (125 MHz) spectral analyses in (DMSO-*d_6_*) gave signals (δ ppm) that match with that previously reported in literature [9]. NMR spectral data are illustrated in Figure S1 as Supplementary Materials data.

### 2.5. Evaluation of the Biological Activity In Vitro

#### 2.5.1. Determination of the Antioxidant Activity using Diphenyl-1-picrylhydrazyl Scavenging Capacity Assay

This was determined using the method previously reported [10,11]. Basically, 200 μL of different concentrations of the isolated compound (PGu) (2.5–150 μg/mL) and 3.8 mL of 60 µg/mL of DPPH solution in methanol were mixed together. The reaction mixture was kept in the dark for 30 min at room temperature followed by measurement of the absorbance at 517 nm. Trolox was used as a positive control. The DPPH^•^: Free radical scavenging activity was determined by applying the following equation:

%Inhibition = [Ac−As/Ac] × 100,(1)

in which Ac: absorbance of control; As: absorbance of sample; and IC_50_ (µg/mL) was obtained from the constructed graph. Additionally, the total antioxidant capacity (TAC) was determined for the compound and compared to Trolox. Total antioxidant activity is expressed as ascorbic acid equivalents. Values are expressed as means ± SEM of three experiments.

#### 2.5.2. Determination of the Anti-Inflammatory Activity using Lipoxygenase Inhibition Assay

Lipoxygenase (LOX) inhibitory potential of PGu was determined using the method previously reported [12,13]. Briefly, a mixture of 1 mL of 0.1 M of sodium borate buffer and 10 µL of 8000 U/mL of soybean LOX was kept with 10 µL of different concentrations of the isolated compound (PGu) (20–280 μg/mL) for 5 min at room temperature in a 1-mL cuvette. This was followed by the addition of 10 µL of 10 mmol of linoleic acid and then the absorbance was measured at 234 nm. Lipoxygenase inhibition was determined from the following equation:

%Inhibition = [Ac–As/Ac] × 100,(2)

in which Ac: absorbance of control; As: absorbance of sample; and IC_50_ (µg/mL) was obtained from the constructed graph. Trolox was used as a positive control. Values are expressed as means ± SEM of three experiments.

### 2.6. Evaluation of the Anti-Epileptic Activity In Vivo

#### 2.6.1. Animals and Animal Treatment

Male Wister rats (150–200 g) were purchased from Nile Company for Pharmaceutical and Chemical industries, Egypt. Approval of the experimental protocol was achieved by the Bioethical and Research committee of Faculty of Pharmacy, Ain Shams University, Cairo, Egypt (Permit no.56). Rats were kept under standard conditions of temperature (24 ± 5 °C) and relative humidity (55 ± 5%) with a light/dark cycle of 12 h each. They were allowed to freely access standard laboratory water as well as laboratory pellets. Moreover, the animals were accommodated to the updated environmental conditions in the housing of the research facility before conducting the experimental study for one week.

#### 2.6.2. Experimental Design of Status Epilepticus in a Rat Model

The animals were distributed randomly into 4 groups, each with 6 animals as follows. In group 1 (control group), animals were treated with the respective vehicles only, administered i.p. with saline, the vehicle of pilocarpine and lithium, and orally administered 0.5% CMC, the vehicle of PGu. However, animals in group 2 were treated with lithium chloride (LiCl)/pilocarpine in a dose of 30 mg/kg b.w. In group 3, lithium chloride (LiCl)/pilocarpine-treated animals orally received 25 mg/kg b.w. PGu. Meanwhile, in group 4, lithium chloride (LiCl)/pilocarpine-treated animals orally received 50 mg/kg b.w. PGu. The protocol was carried out as follows: Animals received LiCl injection (3 mEq/kg b.w., i.p.). After 24 h, the animals were injected with pilocarpine hydrochloride at a dose of 30 mg/kg b.w. In addition, atropine sulfate at a dose of 1 mg/kg b.w. was i.p. administered 30 min before pilocarpine treatment to decrease the cholinergic adverse effects that can occur peripherally. In groups 3–4, the animals were administered two doses of the respective drug 12 h apart, with the second dose administered one hour prior to pilocarpine administration. All the groups administered with pilocarpine were monitored for two hours to detect any behavioral alteration. Seizures scores were assessed according to Racine scale. Briefly, in stage 1, face and vibrissae twitching, ear rubbing with forepaws, and chewing occur. In stage 2, head nodding and unilateral limb clonus is predominant. While in stage 3, limb clonus, and mild whole-body convulsions commonly occur. In stage 4, rearing with bilateral forelimb clonus, tail hypertension, lockjaw, and whole-body convulsions mostly happen. Finally, in stage 5, rearing with whole-body convulsions and falling down with general body rigidity occur [14]. Status epilepticus onset, latency period, and percentages of animals that showed status epilepticus and mortality rates were recorded. Then, each group was splitted into two sets after seizure evaluation, one set for histopathological and immunohistochemical evaluation. However, the other set was used to assess the oxidative stress markers 24 h after status epilepticus induction.

#### 2.6.3. Evaluation of the Biochemical Parameters

##### Estimation of Tissue Malondialdehyde (MDA)

Malondialdehyde (MDA) is considered the main thiobarbituric acid-reactive species (TBARS) and it is indicative of lipid peroxidation. MDA was assessed following the method previously described [15]. Briefly, 0.5 mL of the tissue homogenate, 3 mL of 1% O-phosphoric acid solution, and 1 mL of the 0.6% TBA solution were added, mixed, and then placed in a boiling water bath for 45 min. After cooling, the pink color formed was extracted by *n*-butanol and detected at 2 wave lengths (535 nm and 520 nm), and then the absorbance difference was calculated. MDA levels were expressed as nanomoles per gram tissue (nmol/g tissue).

##### Estimation of Catalase Activity

This was estimated following the instructions of the kit supplied by (Biodiagnostics, Cairo, Egypt) (Aebi, 1984). Briefly, catalase in the tested samples was allowed to react with a measured amount of H_2_O_2_. Stopping of the reaction was done after exactly one minute using a catalase inhibitor. In the presence of peroxidase (HRP), the remaining H_2_O_2_ reacted with 3,5-dichloro-2 hydroxybenzene sulfonic acid (DHBS) and 4-aminophenazone (AAP) to form a chromophore with a color intensity inversely proportional to the amount of catalase in the original sample and its absorbance was measured at 510 nm.

#### 2.6.4. Histological Examination

Samples from rat brains in the various groups were taken by autopsy followed by their fixation for 24 h in 10% formol saline. Tap water was used to wash the samples followed by their dehydration via the use of serial dilutions of different alcohol solutions, which were methanol, ethanol, and absolute ethanol. Then, xylene was used to clear the specimens, then embedded in paraffin and kept for 24 h at 56 °C in a hot oven. Sledge microtome was used to make sections of 4-μm thickness in the prepared paraffin tissue blocks. The prepared tissue sections were gathered on glass slides then deparaffinized and stained with hematoxylin and eosin (H&E) to be examined routinely using a light electric microscope [16]. Scoring was performed by a blinded histologist, using an arbitrary scale, depending on the size and frequency of the lesion.

#### 2.6.5. Immunohistochemical Expression of COX-2 and iNOS Proteins

Tissue sections of a 4-µm thickness were deparaffinized using xylene, then dehydrated using alcohol and heated for 5 min in citrate buffer to retrieve antigen. Then, the manufacturer’s protocol was used to conduct immunohistochemistry staining. Basically, cyclooxygenase-2 (COX-2) monoclonal antibody (Thermo Fisher Scientific, Hemel Hempstead UK, Catalog number RB-9072-P) or iNOS (inducible nitric oxide synthase) (Thermo Fisher Scientific, Hemel Hempstead, UK, Catalog number: RB-9242-R7) or mouse anti-human GFAP (Glial fibrillary acidic protein) (Thermo Fisher Scientific, Hemel Hempstead UK) were incubated separately with the deparaffinized tissue sections after being treated for 20 min with 0.03% H_2_O_2_. The slides were washed by TBS (Tris-Hcl Buffered Saline) and then incubated with the secondary antibody that corresponded to the antigen then the slides were washed again with TBS and kept for 10 min in 0.02% diaminobenzidine (DAB) with 0.01% H_2_O_2_. The slides were counterstained with hematoxylin and examined by a light microscope. Image analysis software (Image J, 1.48a, NIH, Rockville, Maryland, USA) was used for immunohistochemical quantitation of antibodies. For each section, determination of the optical density of stained positive cells was done through 10 different fields.

#### 2.6.6. Statistical Analysis

For parametric data, one-way ANOVA was used to perform the statistical analyses, which were consequently followed by Tukey as a post-hoc test (*p* < 0.05) following the recommendations for data and statistical analyses previously described [17]. Data are represented as mean ± S.E.M. For non-parametric data, Kruskal Wallis followed by Dunn’s test was used for data analysis (*p* < 0.05). Data are presented as median and interquartile range. Mortality rates and the occurrence of seizures were analyzed using the Chi square test for dependence and presented in a contingency table. All statistical analyses and graphs were done using GraphPad Prism software (version 5.01, Inc., 2007; San Diego, CA, USA).

### 2.7. Molecular Modelling Studies

Virtual screening was performed in silico using Discovery Studio 4.5 (Accelrys Inc., San Diego, CA, USA) employing the C-docker protocol. Docking of PGu was performed on 5-lipoxygenase (5-LOX) (PDB ID 3V99, 2.48 Å), which was downloaded from the protein data bank (www.pdb.org). The free binding energies for the highly stable docking poses were calculated in accordance with previously described methods [18,19].

## 3. Results

### 3.1. Isolation and Identification of Pinoresinol-4-*O*-β-d-glucopyranoside

*Prunus domestica* is a rich source of polyphenolic compounds upon extraction with methanol and repeated chromatographic separation and purification. Pinoresinol-4-*O*-β-d-glucopyranoside (PGu) was isolated as one of its predominant constituents. The structure of PGu is illustrated in Figure 1. The structure of the isolated compound was unambiguously determined based upon ^1^H and ^13^C (APT) NMR analyses that matched with that previously reported in the literature [9].

### 3.2. Determination of the Antioxidant Activity In Vitro

**Figure 2:**
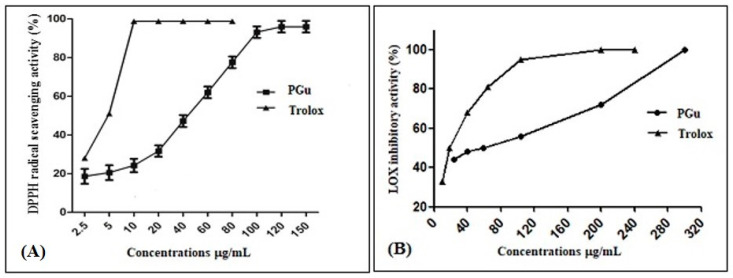
The radical scavenging activity of PGu (Pinoresinol-4-*O*-*β*-d-glucopyranoside) on the (2,2-diphenyl-1-picrylhydrazyl) DPPH^•^ radicals (**A**) and inhibitory effects of PGu on soybean 5-lipoxygenase activity (**B**).

PGu antioxidant activity was determined in vitro using the most commonly used antioxidant assay, which is the diphenyl-1-picrylhydrazyl scavenging capacity assay. PGu displayed a substantial antioxidant activity, with an IC_50_ of 44.2 ± 0.5 µg/mL (Figure 2A) and TAC of 737.7 ± 28.9 µmol/g relative to Trolox, the positive control, which showed an IC_50_ of 4.8 ± 0.4 µg/mL and TAC of 4460.1 ± 28.9 µmol/g.

### 3.3. Determination of the Anti-Inflammatory Activity

This was determined by applying the lipoxygenase inhibition assay, where PGu revealed promising anti-inflammatory activity, with an IC_50_ value of 59.05 ± 0.5 µg/mL relative to Trolox, which displayed an IC_50_ of 18.5 ± 0.5 µg/mL (Figure 2B).

### 3.4. Effect of Pretreatment with Pinoresinol-4-O-β-d-glucopyranoside on LiCl/Pilocarpine-Induced Seizures

**Figure 3:**
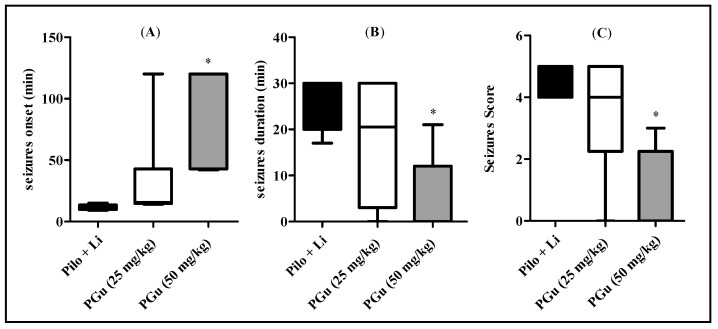
Effects of PGu on pilocarpine + lithium-induced seizures in rats on seizure onset (**A**), seizure duration (**B**) and seizure score (**C**). LiCl was injected i.p. in a dose of 3 mEq/kg b.w. After 24 h, the animals were injected with pilocarpine hydrochloride in a dose of 30 mg/kg b.w. PGu was given in doses 25 and 50 mg/kg twice and the second dose was given 1 h before piloarpine. Data are presented as median (25th, 75th percentile) and analyzed by Kruskal–Wallis and Dunn’s as a post-hoc test. The superscript * indicates significance in relation to the pilocarpine + lithium-treated group at *p* < 0.05.

Pilocarpine intraperitoneal injection resulted in the appearance of a series of behavioral disorders in pilocarpine-treated animals, with the appearance of stereotyped movements. These were represented by rearing, sniffing, and paw licking, which was consequently accompanied by the occurrence of seizures. However, pretreatment with PGu caused a notable amelioration in pilocarpine-induced seizures in a dose-dependent manner as evidenced by the decrease in the animal percentage that developed status epilepticus together with reduced seizure severity in addition to delayed onset of status epilepticus in a significant manner. Regarding the pilocarpine-treated group, all animals (100%) developed seizures, with a mortality rate of 50% after the development of status epilepticus by 24 h. However, pretreatment with 25 mg/kg b.w. PGu slightly alleviated the severity of seizures as manifested by the reduced number of rats that developed seizures (83%), revealing 33% mortality 24 h after the development of status epilepticus. In contrast, pretreatment with 50 mg/kg b.w. PGu significantly ameliorated pilocarpine-induced seizures in which only 33% of rats developed seizures, revealing no mortality after the development of status epilepticus by 24 h. Analysis using the Chi square test showed significant effects on the occurrence of seizures and insignificant effects on mortality rates. Pretreatment with PGu in a dose of 50 mg/kg significantly decreased the seizure duration and score and increased the seizure onset as compared to the pilocarpine-treated animals. However, PGu in a dose of 25 mg/kg did not induce significant changes. PGu given alone at a dose of 25 or 50 mg/kg b.w. had no effect on the measured biochemical parameters. The contingency table showing the effect of PGu on the occurrence of seizures and mortality rates was displayed in Table 1. Meanwhile, the results showing the seizure onset, seizure duration, as well as seizure score were represented in Figure 3.

### 3.5. Effect of Pretreatment with Pinoresinol-4-O-β-d-glucopyranoside on Oxidative Stress Markers

Pretreatment with PGu effectively ameliorated the oxidative stress damage in the cerebral cortex in a dose-dependent manner as evidenced by the reduced level of MDA with a concomitant increase in the catalase activity. Pilocarpine greatly elevated the level of lipid peroxidation in the brain, displaying a 114.7% increase in the MDA level compared to the control group, in addition to reducing the catalase activity in a notable manner, showing 35.4% reduction relative to the control group. Meanwhile, PGu at a dose of 25 and 50 mg/kg b.w. resulted in 13.3% and 24.2% decline in the MDA level, respectively, compared to the pilocarpine-treated group, in addition to increasing the catalase activity by 19.4% and 44.4%, respectively, compared to pilocarpine-treated animals (Table 2).

### 3.6. Effect of Pretreatment with Pinoresinol-4-O-β-d-glucopyranoside on Neuronal Histology

**Figure 4:**
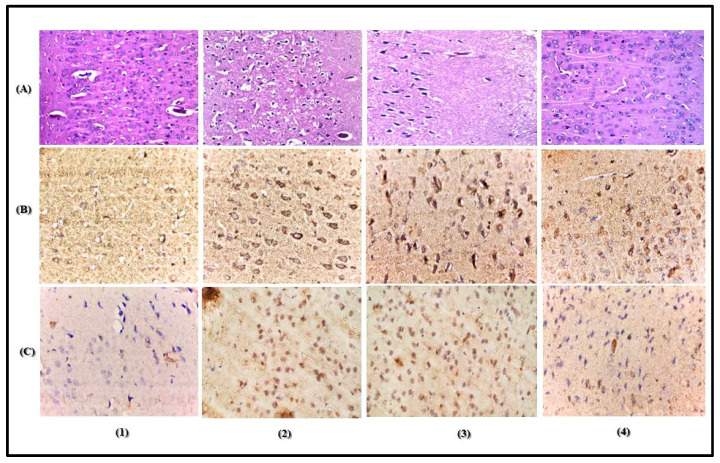
Photomicrographs of cerebral cortex neurons stained with hematoxylin and eosin (**A**), immunohistochemical staining showing Cyclooxygenase-2 (COX-2) (**B**) and Inducible nitric oxide synthase (iNOS) (**C**) expression in animals of the normal control group (**1**), pilocarpine status epilepticus group (**2**), PGu (25mg/kg b.w.) (**3**), and PGu (50 mg/kg b.w.) (**4**) pretreated groups with 40× magnification power.

PGu decreases the neuronal damage in the cerebral cortex as supported by the histopathological examination using hematoxylin and eosin (H & E staining) (Figure 4A). It was clear that animals of the control group displayed normal neurons, which are characterized by a normal alignment and structure in which the nuclei are oval or round, showing clear nucleoli, a regular distribution of chromatin, as well as transparent cytoplasm. However, pilocarpine-treated animals revealed severe neuronal damage evidenced by the appearance of nuclear pyknosis, shrinkage of the cells taking the form of a triangle, degeneration, cellular edema, and vascular congestion in the neurons of the outer and deep cerebral cortex; in addition, some nuclei appeared in a crescent form. However, pretreatment with PGu in a dose of 25 mg/kg b.w. did not show any significant neuronal protection, where the cerebral cortex showed nuclear pyknosis and degeneration in the neuronal cells. Meanwhile, it is obvious from Figure 4A that pretreatment with PGu in a dose of 50 mg/kg b.w. effectively attenuated neurodegeneration triggered by seizure induction as there was no histopathological alteration. The normal histological structure of the cerebral cortex neurons was recorded and approached that of the normal control group. Additionally, a table representing the scoring of histological changes was added to better illustrate the histological variations among the different groups (Table 3).

### 3.7. Effect of Pretreatment with Pinoresinol-4-O-β-d-glucopyranoside on Neuroinflammation Manifested by COX-2, iNOS, and GFAP Immunohistochemical Expression

**Figure 5:**
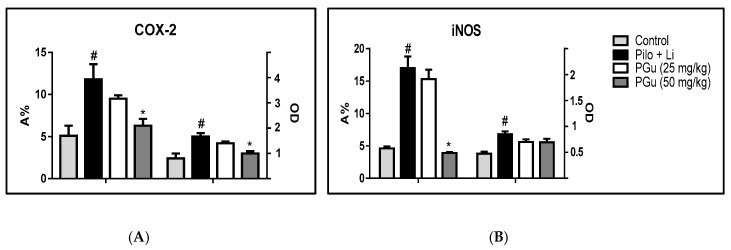
Quantitative image analysis for COX-2 (**A**) and iNOS (**B**) immunohistochemical staining expressed as optical densities (OD) across 7 different fields for each rat section. LiCl was injected at a dose of 3 mEq/kg b.w. i.p. After 24 h, the animals were injected with pilocarpine hydrochloride at a dose of 30 mg/kg b.w. PGu was given doses of 25 and 50 mg/kg twice and the second dose was given 1 h before pilocarpine. Data are presented as mean ± S.E. The superscripts # and * indicate significance in relation to the control and pilocarpine + lithium-treated groups, respectively, at *p* < 0.05.

**Figure 6:**
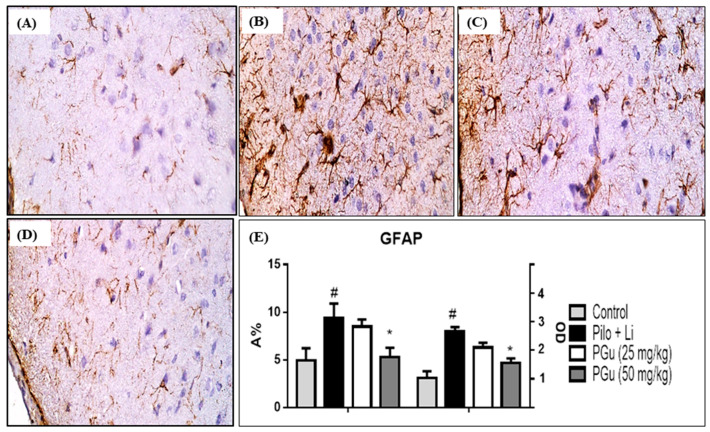
Photomicrographs of cerebral cortex neurons stained with immunohistochemical staining showing Glial fibrillary acidic protein (GFAP) expression in animals of the normal control group (**A**), pilocarpine status epilepticus group (**B**), PGu (25mg/kg b.w.) (**C**), and PGu (50 mg/kg b.w.) (**D**) pretreated groups with 40× magnification power while (**E**) shows quantitative image analysis for GFAP immunohistochemical staining expressed as optical densities (OD) across 7 different fields for each rat section. LiCl was injected at a dose of (mEq/kg b.w., i.p.). After 24 h, the animals were injected with pilocarpine hydrochloride at a dose of 30 mg/kg b.w. PGu was given in doses of 25 and 50 mg/kg twice and the second dose was given 1 h before pilocarpine. Data are presented as mean ± S.E. The superscripts # and * indicate significance in relation to the control and pilocarpine + lithium-treated groups, respectively, at *p* < 0.05.

**Figure 7:**
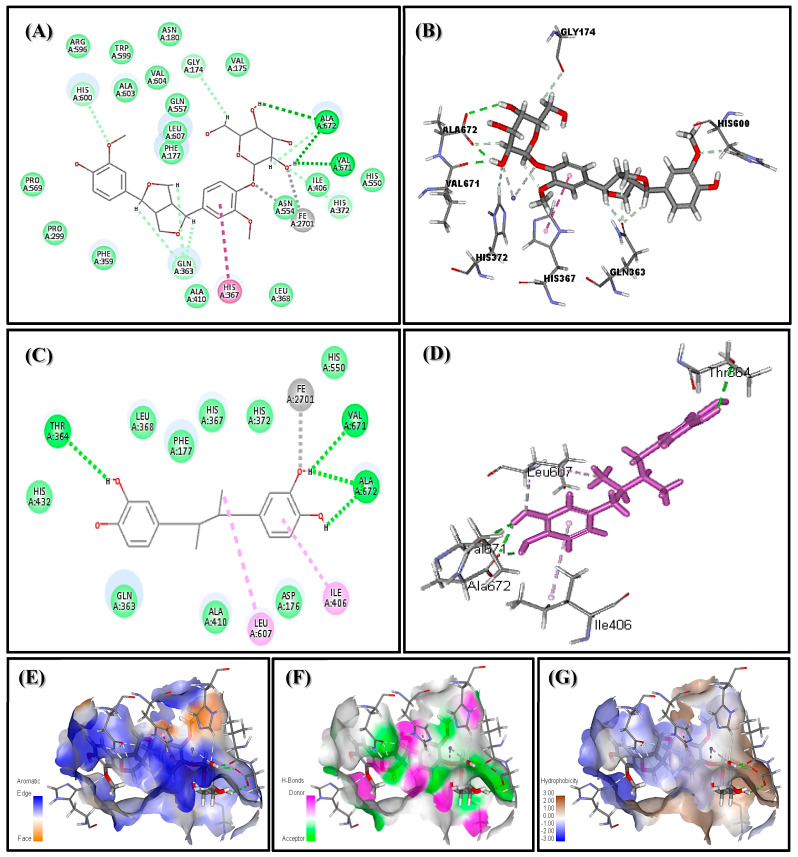
2-D (**A**) and 3-D (**B**) binding modes of PGu and 2-D (**C**) and 3-D (**D**) binding modes of nordihydroguaiaretic in 5-lipoxygenase (5-LOX) active sites and the presence of PGu in the active pocket of the receptor showing the aromatic regions (**E**), regions of hydrogen bond formation (**F**), as well as hydrophobicity regions (**G**) in 5-LOX.

Immunohistochemical staining was used to assess the expression levels of the inflammatory biomarkers COX-2 and iNOS in the cerebral neurons. The control group displayed a reduced level of immunostaining for both markers; meanwhile, pilocarpine-treated animals showed significant elevation in both COX-2 and iNOS by an estimated 108% and 79%, respectively, in its assessed optical density (OD) and 131% and 269%, respectively, in the area percent of stained cells (A%) compared to the control group. PGu in a dose of 25 mg/kg b.w. insignificantly reduced the elevated COX-2 and iNOS expression by 15% and 17%, respectively, as expressed by the OD and 19% and 16%, respectively, as expressed in A%. In contrast, pretreatment with PGu in a dose of 50 mg/kg b.w. significantly declined the elevated COX-2 by 40% and insignificantly decreased iNOS expression by 18% as expressed by OD and significantly reduced the A% of both COX-2 and iNOS stained cells by 46% and 77%, respectively. The immunohistochemical staining was determined via quantitative assessment of the optical density of the stained areas using the image analysis software and is represented in Figure 4B,C and Figure 5. Additionally, to better explain the potential effect of PGu on glial cells, immunohistochemical staining of the astrocyte-specific protein, glial fibrillary acid protein (GFAP), the marker of astrocytosis, was assessed (Figure 6). Pilocarpine significantly elevated the expression of GFAP compared to the control group, estimated by 1.6-fold and 91% in its assessed OD and A%, respectively. However, PGu at a dose of 25 mg/kg b.w. substantially reduced the elevated GFAP expression by 25% and 9.5%, respectively, as expressed by the OD and A%, respectively, relative to the Li/pilocarpine-treated group. In contrast, at 50 mg/kg b.w., PGu effectively declined the elevated GFAP expression as evidenced by its faint immunoreactivity, as revealed in Figure 7, estimated by 41% and 37% as expressed by the OD and A%, respectively, with respect to the Li/pilocarpine-treated group.

### 3.8. Molecular Modelling Studies

In silico molecular modelling of PGu was done on 5-LOX, which is implicated in neuroinflammation and accompanies epileptic seizures, in an effort to confirm the in vitro as well as the in vivo anti-inflammatory potential of PGu. PGu revealed a pronounced fitting within the active site of 5-LOX as displayed from the value of its binding free energy (∆G) of −65.05 kcal/mol, exceeding that of nordihydroguaiaretic acid (NDGA), a potent anti-inflammatory agent, which revealed (∆G) of −61 kcal/mol. Two-dimensional and 3-D binding modes of PGu within 5-LOX’s active pocket are represented in Figure 6. PGu effectively inhibits 5-LOX by binding to its active sites tightly through the formation of three conventional hydrogen bonds between the hydroxyl functional groups in the glucose moiety and the ALA:672 and VAL:671 amino acid residues existing at the active site in addition to the formation of π-bond between the aromatic benzene ring and HIS:367 residue. Besides, many Van der Waals interactions occur between the different functional groups containing oxygen in the aglycones portion of PGu and HIS:600, GLY:174, ALA:672, ILE:406, as well as GLN:363 amino acid residues at the active sites. Furthermore, a strong metal acceptor bond formed between the molecule and iron existing in the active site, Fe2071. Meanwhile, nordihydroguaiaretic acid (NDGA) forms four H-bonds with VAL: 671, ALA: 672, and THR: 3 64; two π-bonds with LEU: 607 and ILE: 406; in addition to the strong metal acceptor bond formed between the molecule and iron existing in the active site, Fe2071. Thus, it seems that the metal acceptor bond with Fe2071, and the H- bonds with ALA: 672 and VAL: 671 are crucial for effective enzyme inhibition, as illustrated in Figure 7.

## 4. Discussion

The induction of seizures utilizing pilocarpine combined with lithium has been widely accepted as an animal model possessing many criteria of human epilepsy manifested by an electroencephalographic pattern with the consequent behavioral as well as morphological pattern. When pilocarpine is intraperitoneally injected, it triggers signs of status epilepsy that seriously damage neurons and cause excitotoxic damage [20]. Oxidative stress and inflammation are highly correlated to the occurrence of convulsions, which represents the signs of certain types of epilepsy [21,22,23].

In the foregoing study, it was found that the pretreatment of rats with PGu, a dietary lignan glucoside obtained from the prunes of *Prunus domestica,* effectively ameliorates the seizures triggered by pilocarpine in a dose-dependent manner, as manifested by retardation in seizure onset, reduction in the number of rats that developed seizures with a concomitant reduction in its severity, and enhancement in the animal’s survival after seizure exposure.

Besides, oxidative stress greatly worsens the state of epilepsy, accelerating its occurrence as well as its progression, and is strongly correlated with excitotoxicity [24]. Hence, antioxidants can effectively prohibit seizure initiation and ameliorate neuronal damage. Results showed that pretreatment of rats with PGu effectively ameliorates oxidative stress damage in the cerebral cortex in a dose-dependent manner as evidenced by the reduced level of MDA with a concomitant increase in catalase activity, which acts as a natural reactive oxygen species scavenger. PGu is a lignan having a furofuran skeleton with phenolic groups, which acts as a potent antioxidant via the scavenging of free radicals.

These results were further consolidated by the in vitro antioxidant activity assessment using the DPPH scavenging capacity assay displaying an IC_50_ of 44.2 µg/mL. Additionally, lignans significantly inhibit the ability of many transition elements, exemplified by copper and iron triggering free radical chain reactions via the formation of complexes through their adjacent –OH moieties or extra chelating groups [25,26].

Regarding the histological manifestation of the neuronal structure, it was found that pilocarpine exerts a massive cellular destruction that effectively participates in epileptogenesis. It induces neuronal damage, as evidenced by the appearance of nuclear pyknosis, shrinkage of the cells taking the form of triangle, degeneration, and cellular edema. The pretreatment of rats with PGu alleviated neurodegeneration caused by pilocarpine, as manifested by the existence of a normal histological structure of the cerebral cortex neurons, particularly at a dose of 50 mg/kg b.w.

Besides, the pretreatment of rats with PGu counteracted the inflammation triggered by pilocarpine/Li treatment. It is noteworthy to highlight that a previous study was carried out on PGu, and confirmed its antiviral activity versus influenza (H1N1) virus via its anti-inflammatory potential, as manifested by the prohibition of nuclear factor-κB stimulation, and inhibition of the p38 mitogen-activated protein kinase (MAPK) and Protein Kinase B (PKB/AKT) signaling pathways. Moreover, PGu effectively inhibited influenza A virus subtype H1N1 H1N1 virus-stimulated proinflammatory mediators’ expression, comprising monocyte chemo-attractant protein 1, interleukin (IL)-6, IL-8, and tumor necrosis factor-α [27]. Furthermore, it was reported that epileptogenesis is exaggerated by the continual production of proinflammatory mediators caused by chronic inflammation as well as seizures, leading to elevated excitation in neurons, nerve cell damage, and dysfunctional BBB [3]. PGu exerted a notable anti-inflammatory activity and effectively decreased the elevated COX-2 and iNOS expression in a dose-dependent manner, as manifested by the immunohistochemical expression. COX-2 is an enzyme that greatly affects nerve cell damage caused by seizures via oxidative stress, and excitotoxicity caused by glutamate or by prostaglandins’ neurotoxic potential. COX-2 inhibitors are considered as potent therapeutic approaches in the alleviation of inflammatory neurodegenerative pathways [28]. Meanwhile, the expression of iNOS in sensitive brain areas greatly elevates the susceptibility to seizures [29]. PGu’s anti-inflammatory potential was further supported by in vitro study, where PGu effectively inhibited 5-LOX of soybean, with an IC_50_ value of 59.05 µg/mL. The anti-inflammatory potential of PGu was further confirmed using molecular modelling studies on 5-LOX’s active site, which is implicated in the sparking and progression of inflammation [30]. 5-LOX is also entangled in many pathological disorders comprising convulsion as well as many neurodegenerative ailments that could be partly explained by the upregulation of iNOS expression [29]. PGu effectively inhibits 5-LOX by binding tightly to its active sites through the formation of three hydrogen bonds, π-bond, many Van der Waals interactions, and a metal acceptor bond at the active sites (Figure 7). This confirms the anti-inflammatory activity of PGu and its role, in turn, in alleviating convulsions and neuroinflammation associated with epilepsy. It is worthy to highlight that many lignans exerted a promising neuroprotective potential, where (+)-pinoresinol and herpetol displayed potent inhibitory effects on the cell death of the glutamate-induced HT22 (Mouse Hippocampal Neuronal Cell Line) cells and on NO production in (lipopolysaccharide) LPS-induced mouse macrophages RAW264.7 cells in a dose-dependent manner. They exerted a protective behavior estimated by 86.0% and 83.9% inhibition versus glutamate-induced cell death at a concentration of 40 μM, which is superior to the protective effect elicited by the positive control, Trolox [31]. Besides, neolignans represented by honokiol, obovatol, and nethylhonokiols were reported to possess neuroprotective effects that probably offer promising drug leads for the alleviation and prohibition of neuronal disease [32].

## 5. Conclusions

In the foregoing study, it was concluded that pinoresinol-4-*O*-β-d-glucopyranoside exerted potent neuroprotective activity as manifested by the alleviation of lithium/pilocarpine-induced epileptic seizures and neuroinflammation in rats. This pronounced activity greatly relies upon the antioxidant and anti-inflammatory activity of PGu as evidenced by the in vitro study, which was further consolidated by the in vivo results. The presence of the furofuran skeleton with the existence of phenolic groups mainly accounts for the promising activity of the compound. Thus, this lignan glucoside could perfectly serve as a potent therapeutic agent for the relief of epileptic seizures, which is highly welcomed by a large category of patients owing to its natural origin. However, additional clinical trials are recommended to declare the possibility of its utilization in human disorders. Future studies to better understand the pharmacokinetic–pharmacodynamic correlation are to be carried out. Additionally, other seizure models, such as (pentylenetetrazole) PTZ or (electroconvulsive seizure) ECS, are recommended to ascertain the obtained results.

## Figures and Tables

**Table 1:** Contingency table showing the effect of PGu on the occurrence of seizures and mortality rates.

		Seizure Occurrence			Mortality		
Group	No of Animals and	Convulsed	Non-Convulsed	%Convulsed	Survived	Dead	%Mortality
**Control**	6	0	6	0%	6	0	0%
**Pilo + Li**	6	6	0	100%	3	3	50%
**PGu (25 mg/kg)**	6	5	1	83%	4	2	33%
**PGu (50 mg/kg)**	6	2	4	33%	6	0	0%

**Table 2:** Effect of PGu on the oxidative stress biomarkers (MDA) and (CAT) in pilocarpine-induced seizures in a rat model.

Group	MDA (nmol/g tissue)	% Change	Catalase (U/g tissue)	%Change
**Control**	58.7 ± 3.3	−53.41% ^b^	44.6 ± 1.9	+54.86% ^b^
**Pilo + Li**	126 ^#^ ± 6.3	+114.7% ^a^	28.8 ^#^ ± 0.7	−35.4% ^a^
**PGu (25 mg/kg)**	109.3 ± 5.8	−13.3% ^b^	34.4 ± 1.8	+19.4% ^b^
**PGu (50 mg/kg)**	95.5 ^*^ ± 5.9	−24.2% ^b^	41.6 ^*^ ± 1.3	+44.4% ^b^

Catalase activity and MDA levels data are presented as mean ± SEM (*n* = 6) and analyzed by one-way ANOVA then Tukey as a post-hoc test. The superscripts # and * indicate significance in relation to the control and pilocarpine + lithium-treated groups, respectively, at *p* < 0.05. ^a^ % Change relative to the control group; ^b^ % Change relative to pilocarpine-treated groups, at *p* < 0.05.

**Table 3:** Scoring of the histological changes among different groups.

Histopathological Parameter	Control	LiCl/pilo	PGu (25 mg/kg)	PGu (50 mg/kg)
**Nuclear pyknosis**	−	+++	+++	−
**Edema**	−	+++	++	+
**Vascular congestion**	−	+++	++	+
**Degeneration**	−	+++	+++	−

(−) = null; (+) = mild; (++) = moderate; (+++) = severe.

## Supplementary Materials

The following are available online at https://www.mdpi.com/2076-3921/9/7/575/s1. Figure S1: NMR data of Pinoresinol-4-*O*-β-d-glucopyranoside.
