# Supplementary material for: A Potent Lignan from Prunes Alleviates Inflammation and Oxidative Stress in Lithium/Pilocarpine-Induced Epileptic Seizures in Rats
Source: Antioxidants (Basel). 2020 Jul 2;9(7):575. doi: 10.3390/antiox9070575 (PMC7402155; doi:10.3390/antiox9070575)
Supplement: Supplementary file 1 [file antioxidants-09-00575-s001.pdf]

Supplementary data

A potent lignan from prunes alleviates inflammation and oxidative stress in lithium/pilocarpine-induced epileptic seizures in rats

Fig. S1: NMR data of Pinoresinol-4-*O*-*β*-D-glucopyranoside

Fig. S1: NMR data of Pinoresinol-4-*O*-*β*-D-glucopyranoside

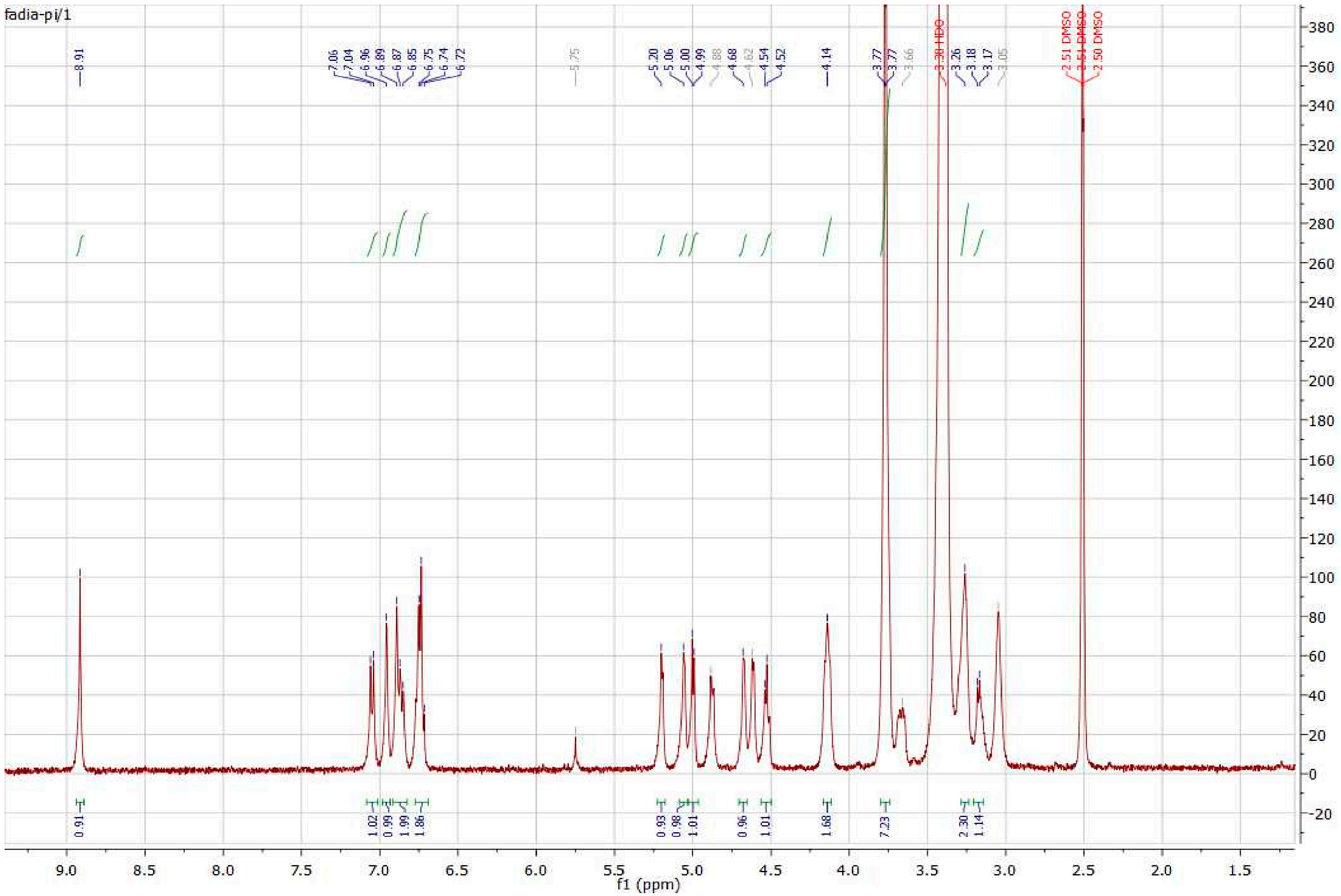

***[1]*H-NMR data of Pinoresinol-4-*O*-*β*-D-glucopyranoside**

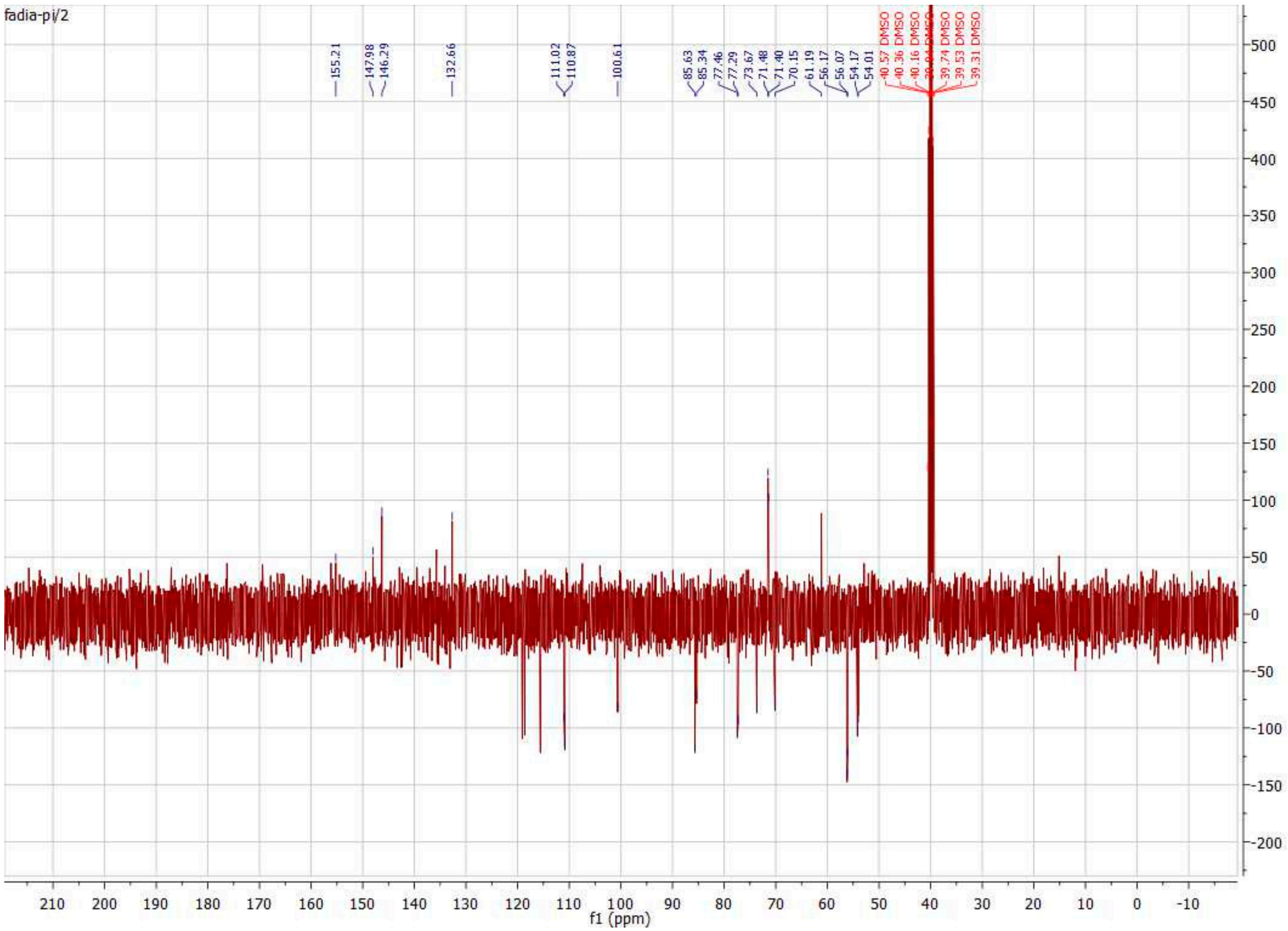

APT-NMR data of Pinoresinol-4-*O*-*β*-D-glucopyranoside
